# Training the Hand

**Published:** 1899-10

**Authors:** 


### Training the Hand.

While there are many professions in which the artisans have to acquire greater manual dexterity than is common in the practice of the surgical art, the cultivation of the *tactus eruditus* is of far greater import in our work than seems generally thought to be the case by surgeons themselves. They frequently neglect this branch of their education to a very extraordinary extent, probably because, in this age of anesthesia, they are able to operate slowly, and, if they know what is to be done and how to do it, manage to obtain good results. But in order to know what is to be done one must be a good diagnostician, and without skilled hands and fingers that have mastered the art of palpating until every nervous papilla is an additional eye with a clear vision, the surgeon remains a mechanic, capable of executing, but incompetent, in many instances, to decide upon the advisability of execution. The surgeons of olden times, with their rapid operations, possessed greater skill than we do. It is said of Liston that he could take up a roast goose on a fork, hold it in the air, and with his right hand so dexterously ply his knife as to carve it in masterful fashion. It is probable that he knew nothing about feeling the appendix, but it is certain that he could have learnt to do it in a few minutes. The only way to acquire skill is to constantly practice. Every time the abdomen is examined, for instance, the surgeon should make it a habit to feel the appendix, to make out the lower border of the liver, to palpate the kidneys, and to examine for gall-stones. Patients never object to a thorough examination as long as no unnecessary pain is inflicted, and in some instances unsuspected conditions will thus be revealed. The practitioner should never allow a chance to further train his hands to escape him, for the results will more than compensate him for his trouble.—*International Journal of Surgery.*

